# Genome Wide Structural Variants Provide Insights Into Population Structure and Genetic Divergence in Pacific White Shrimp ( *Penaeus vannamei* ) Breeding Populations

**DOI:** 10.1111/eva.70312

**Published:** 2026-08-24

**Authors:** Mingyang Zhao, Hao Wang, Mingxuan Teng, Hongyu Lv, Baojun Zhao, Mengqiang Wang, Zhenmin Bao, Qifan Zeng

**Affiliations:** ^1^ Hainan Key Laboratory of Tropical Aquatic Germplasm (Hainan Seed Industry Laboratory), Sanya Oceanographic Institution Ocean University of China Sanya China; ^2^ MOE Key Laboratory of Marine Genetics and Breeding, Shandong Key Laboratory of Marine Seed Industry (Preparatory), and Qingdao Institute of Maritime Silk Road (Qingdao Institute of Blue Seed Industry) Ocean University of China Qingdao China; ^3^ Southern Marine Science and Engineering Guangdong Laboratory (Guangzhou) Guangzhou China

**Keywords:** genetic divergence, *Penaeus vannamei*, population structure, selective sweep, structural variations

## Abstract

Structural variants (SVs) are a major yet underused source of adaptive variation in aquaculture. We built a genome‐wide SV atlas for 180 *Penaeus vannamei* from six commercial breeding populations and discovered 1,159,046 SVs, with uneven chromosomal distributions and multi‐type hotspots. Over 63.53% of SVs overlapped repeats—especially simple sequence repeats, DNA transposons, and LINEs. SV and SNP densities were highly correlated. Across populations, 482 k SVs were shared and 145,623 were singletons; the fraction of deletions increased from shared to singleton classes. BMK and KH harbored more singletons than SIS, RH, and CP, indicating greater divergence. PCA and ADMIXTURE recovered three major clusters and revealed the substructure in RH, mirroring SNP analyses. Selection scans identified 78–193 sweep windows per population encompassing 38–161 candidate genes. These genes were predominantly enriched in population‐specific processes such as chromatin regulation, meiotic recombination, membrane‐associated functions, suggesting that structural variants may contribute to divergence in reproductive, metabolic, and structural pathways across breeding programs. Nevertheless, 10 genes showed parallel signals in over 3 populations; many carry short deletions likely affecting regulatory or coding elements. Together, these results show that genome architecture and domestication jointly shape the shrimp SV landscape; that SVs alone robustly resolve population history; and that a small set of recurrent, deletion‐bearing regulatory genes may underpin convergent improvement. The SV map and candidate loci provide diagnostic markers for germplasm tracing and candidate loci for marker‐assisted or genomic selection in *P. vannamei* breeding.

## 1 Introduction

Selective breeding of the Pacific white shrimp ( *Penaeus vannamei* ) over the past few decades has led to the establishment of multiple commercial lines with improved growth, survival, and disease resistance (Campos‐Montes et al. 2023; Ren et al. 2020; Tan et al. 2019; Zeng et al. 2022). These breeding populations have distinct genetic backgrounds due to separate founder events and selection histories (Medrano‐Mendoza et al. 2023; Wang et al. 2022). Understanding the genetic basis of their adaptation is crucial for both evolutionary insight and practical applications in aquaculture (Max et al. 2025; Wang et al. 2017). Previous genomic studies in *P. vannamei* have primarily utilized single nucleotide polymorphisms (SNPs) to assess genetic diversity, population structure, and selection (Prithvisagar et al. 2021; Wang et al. 2022). However, SNPs represent only one class of genetic variation. Structural variants (SVs) can have profound phenotypic effects and may capture a different spectrum of adaptive variation not detectable by SNP analyses (Collins and Talkowski 2025; Huang et al. 2024).

SVs contribute substantially to genetic diversity and evolution, but they have remained underrepresented in population genomics, especially for non‐model species, due to technical challenges in detection (Mahmoud et al. 2019; Pokrovac and Pezer 2022). In *P. vannamei* , which has a large and repeat‐rich genome, SVs are expected to be abundant (Peng et al. 2023; Zhang et al. 2019). The shrimp genome assembly is notable for an extremely high content of simple sequence repeats (SSRs) and transposable elements (TEs), which not only complicate genome assembly but also likely facilitate structural rearrangements (Yuan et al. 2021). Repetitive sequences can promote SV formation through mechanisms such as unequal crossing‐over or transposon insertions (Fedoroff 2012; Yang et al. 2013). Thus, characterizing SVs in this species may reveal mutational processes and regions of the genome particularly prone to change. At the same time, structural changes in gene‐rich regions may be subject to strong purifying selection (Pös et al. 2021). Examining the distribution of SVs relative to genomic features can shed light on how genome architecture influences the patterns of genetic variation (Collins and Talkowski 2025; Yang et al. 2013).

Beyond their intrinsic interest, SVs are increasingly recognized as important drivers of phenotypic diversity and adaptation (Gong et al. 2025; Karageorgiou et al. 2024). In domesticated animals and plants, SVs have been implicated in traits ranging from morphology to stress tolerance (Alonge et al. 2020; Dai et al. 2025). In *P. vannamei* , emerging evidence suggests SVs play roles in economically important traits—for example, recent work has linked certain SVs to differences in reproductive output in shrimp (Huang et al. 2024). Because selective breeding imposes strong artificial selection and often involves population bottlenecks, it can leave distinct signatures on the genome (Andersson and Purugganan 2022). Theory predicts that small effective population size during domestication can relax the efficacy of negative selection, allowing mildly deleterious mutations to persist or even fix via drift and hitchhiking (Bortoluzzi et al. 2020; Makino et al. 2018). Indeed, studies in other domesticated species have found that population bottlenecks associated with domestication led to an increased burden of deleterious variants, as these mutations piggybacked on regions under positive selection (Bortoluzzi et al. 2020; Yang et al. 2017). It is therefore of interest to investigate whether the breeding histories of *P. vannamei* lines have resulted in the accumulation of particular types of SVs within individual stocks.

In this study, we conducted a comprehensive analysis of SVs of 180 *P. vannamei* individuals from six major breeding populations. The objectives were as follows: (1) construct a high‐resolution SV atlas and characterize the distribution of SVs across the genome in relation to chromosomes and genomic elements; (2) evaluate genetic differentiation and relationships among the populations using SV markers, and compare these results with SNP‐based inferences; and (3) identify genomic regions and genes showing evidence of artificial selection based on inter‐population SV frequency differences, and to highlight candidate genes that may underlie adaptive divergence or convergent selection across the breeding programs. By integrating these analyses, we aim to expand the knowledge of how structural genomic variation has been shaped by and contributes to evolutionary processes in a domestication context, and to provide a genomic resource for future studies on shrimp breeding and genetic resource conservation. Our work represents one of the first genome‐wide SV studies in an aquaculture species, and it underscores the importance of looking beyond SNPs to fully capture the genomic changes during selective breeding.

## 2 Materials and Methods

### 2.1 Data Collection and SNP Detection

The whole‐genome resequencing data for 180 *P. vannamei* individuals used in this study were obtained from the publicly accessible Aquaculture Molecular Breeding Platform, as originally reported by Wang et al. (2022) and Zeng et al. (2022). These data cover six commercially farmed lines—Renhai No. 1 (RH), Kehai No. 1 (KH), Benchmark Genetics (BMK), Charoen Pokphand (CP), Shrimp Improvement Systems (SIS), and Top Aquaculture Technology (TA)—and were generated as 300‐bp paired‐end reads on the MGI DNBSEQ‐T7 platform, with an average depth of approximately 18.7× per sample. In the original study, sequence reads were mapped to the *P. vannamei* reference genome using BWA (v2.3.4.1), and the resulting alignments were processed with SAMtools and Picard2 for sorting, indexing, and read‐group assignment, followed by duplicate removal with GATK (v4.1). Variant discovery was carried out with GATK HaplotypeCaller in GVCF mode, and joint genotyping was performed using GenotypeGVCFs. SNPs were subsequently filtered by applying thresholds for GQ, QD, FS, MQ, ReadPosRankSum, SOR, and MQRankSum to retain high‐confidence variants. Additional filtering with VCFtools (v0.1.16) removed SNPs with multiple alleles, low minor allele frequency (MAF < 0.05) to reduce noise and improve the robustness of downstream population genetic analyses, as well as excessive missing genotypes (max‐missing < 1) (Table S1) (Danecek et al. 2011). For analyses requiring reduced SNP density, genome‐wide LD decay was estimated using PopLDdecay, and the LD decay distance was calculated as 260 kb (*r* ^2^ = 0.0068, corresponding to the bottom 5% of the genome‐wide LD distribution) (Figure S1). Based on this threshold, linked loci were removed from the SNP dataset using PLINK with adjusted ‐‐indep‐pairwise parameters, yielding a final set of 12,097 SNPs for downstream analyses.

### 2.2 Structure Variants Detection

SV calling was performed based on the BAM files obtained from SNP processing, to ensure comparability between the SNP and SV analyses (Zeng et al. 2022). Individual short SVs were identified independently in each of the 180 samples using Delly (v1.0.3) with default recommended settings, yielding a raw dataset of 1,159,046 SVs (Rausch et al. 2012). Repeat elements were annotated with RepeatMasker (v4.0.7) (Nishimura 2000). The resulting dataset was filtered using VCFtools (v0.1.16) to remove SVs with high missingness (‐‐max‐missing 0.5) or low minor allele frequency (‐‐maf 0.05), ensuring the retention of well‐represented SVs for subsequent analyses, resulting in a final set of 295,542 SVs (Table S1) (Danecek et al. 2011). SVs were classified into four categories according to their prevalence among the six breeding lines: “Shared” (present in all six), “Low frequency” (detected in four or five lines), “Rare” (found in two or three), and “Private” (unique to a single breeding line).

### 2.3 SNP and SV Density Analysis

To evaluate the genome‐wide relationship between SNP and SV densities, Bedtools (v2.25.0) was used to calculate the number of variants within non‐overlapping 10 Mb windows across all 44 linkage groups (Quinlan and Hall 2010). SNP counts were derived from the whole‐genome SNP dataset, whereas SV counts were obtained from the unfiltered SV dataset. Variant densities per window were subsequently analyzed in R (v4.4.2) using a generalized linear model (GLM) with a quasipoisson error distribution and identity link function, with SNP density modeled as the independent variable and SV density as the dependent variable. Model residuals were further extracted and visualized as a 10 Mb window‐based heatmap to identify genomic windows in which SV density deviated from the level expected based on SNP density, thereby allowing the identification of regions with an excess or deficit of SVs relative to SNP‐based expectations. Differences in gene content, repetitive elements, and other genomic features within these regions, as well as their potential underlying causes, were subsequently investigated.

### 2.4 Genetic Structure and Diversity Analysis

Parallel analyses were conducted using the thinned SNP dataset and the filtered SV dataset to assess population structure and genetic diversity. Population ancestry was inferred with ADMIXTURE (v1.3.0) (Alexander et al. 2009). Principal component analysis (PCA) was performed using PLINK (v1.9) and GCTA (v1.94.0) to capture the major axes of genetic variation (Purcell et al. 2007; Yang et al. 2011).

### 2.5 Selective Sweep Analysis

Pairwise *F*_ST_ values and the ratio of nucleotide diversity (*θ*_*π*_) between breeds were calculated using 50 kb sliding windows with a 10 kb step size. Genomic regions exhibiting significant divergence in polymorphism spectra were considered putatively under selection. Empirical thresholds were set at the top 2% of *F*_ST_ values and the top 5% of the highest or lowest *θ*_*π*_ ratios (Li et al. 2018; Wang et al. 2021, 2022).

In addition to the *F*_ST_–*θ*_*π*_ approach, XP‐EHH (Sabeti et al. 2007), analysis was performed using selscan (v3.0.1) to provide haplotype‐based support for the identification of candidate selective sweep regions (Rahman et al. 2025). Prior to XP‐EHH analysis, SV genotypes were phased using Beagle (v5.5) (Browning et al. 2021). For each focal breed, phased biallelic SVs were extracted from the whole‐genome SV dataset, with the focal breed used as the target population and the remaining breeds combined as the reference population. A map file containing chromosome ID, SV ID, genetic distance approximated from physical position, and physical position was prepared for each chromosome.

XP‐EHH was calculated separately for each chromosome using the ‐‐xpehh option in selscan. The raw XP‐EHH scores were normalized using the norm utility implemented in selscan. Normalized XP‐EHH values were then summarized in 50 kb windows with a 10 kb step size, consistent with the window size used in the *F*_ST_–*θ*_*π*_ analysis. Windows were ranked according to their mean XP‐EHH values, and an empirical threshold of the top 10% was applied to define candidate XP‐EHH regions (Rodrigues et al. 2026).

To reduce potential false‐positive signals, candidate regions identified by XP‐EHH were intersected with those detected by the *F*_ST_–*θ*_*π*_ approach. Genomic regions supported by both differentiation/diversity‐based and haplotype‐based evidence were retained as high‐confidence candidate selective sweep regions for subsequent gene annotation and enrichment analysis.

Candidate genes under selection were defined as those located within or overlapping these regions. Gene Ontology (GO) enrichment analysis of candidate genes was performed using the EnrichPipeline, based on Fisher's exact test. GO terms with *p* < 0.01 were considered significantly enriched (Chen et al. 2011).

## 3 Results

### 3.1 Structural Variant Discovery and Genome‐Wide Distribution

Whole‐genome resequencing and read alignment to the reference shrimp genome yielded a comprehensive set of structural variants (SVs). In total, 1,159,046 SVs were identified across the 180 samples, comprising five SV types: 596,839 deletions (DEL), 63,786 insertions (INS), 114,477 duplications (DUP), 28,616 inversions (INV), and 355,328 translocation breakends (BND) (Table 1). This corresponds to an average of approximately 6439 SVs per individual genome. The sizes of the identified SVs ranged from the detection limit of approximately 15 bp to 999 kb, with most variants representing small or intermediate‐size events. More than 57% of all SVs were small indels, including insertions and deletions, and more than half of these indels were shorter than 500 bp. Only 2.72% of SVs exceeded 10 kb in length, indicating that short‐read resequencing mainly detected small and medium‐sized SVs (Table S2, Figure S2).

**TABLE 1:** Summary of structural variants identified in this study.

SV	Counts	Length (bp)		
		Max	Mean	Median
INV	28,616	999,246	32,818.23	1472
INS	63,786	91	32.94	29
DEL	596,839	998,787	1858.58	53
DUP	114,477	999,308	9200.56	540
BND	355,328	—	—	—

**FIGURE 1:**
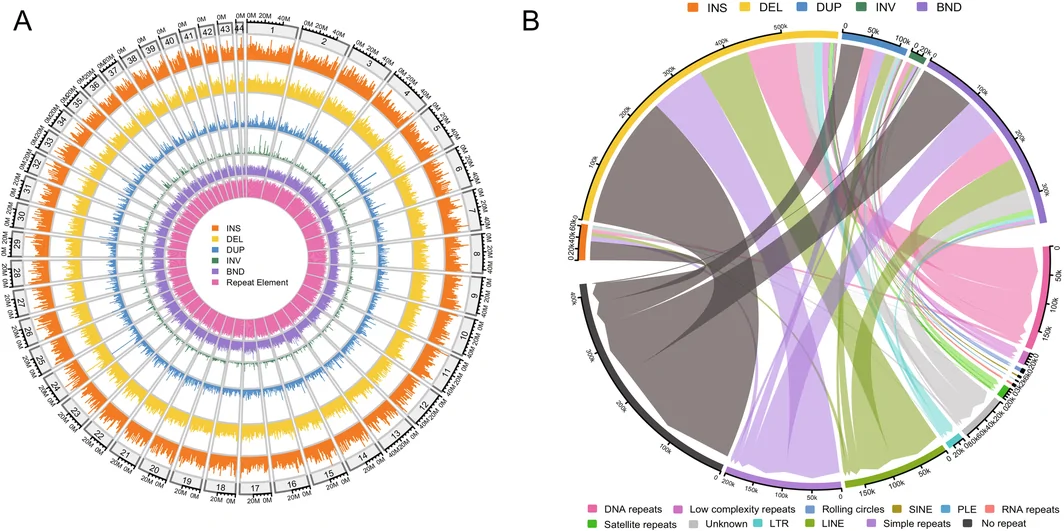
Genome‐wide identification of structural variations in 180 individuals from six populations. (A) The outermost layer shows the chromosomal framework. From the outside to the inside, the next five layers represent the distributions of five types of structural variants (INS, DEL, DUP, INV, and BND). The innermost layer depicts the distribution of repetitive elements. All data were summarized using 1‐Mb windows. (B) The types of repetitive elements annotated for the five classes of structural variants.

SVs were unevenly distributed across the genome. Chromosome 1 contained the largest number of SVs, with 32,853 events, whereas chromosome 44 contained the fewest, with 4226 events (Figure 1A). After normalization by chromosome length, SV density still varied markedly among chromosomal regions. Several genomic regions showed local accumulation of multiple SV types. For example, SV‐rich regions were observed on chromosome 1 at 29.13–29.98 Mb, chromosome 3 at 20.00–20.99 Mb, chromosome 22 at 20.09–20.99 Mb, and chromosome 30 at 3.00–3.99 Mb (Figure 1A).

**FIGURE 2:**
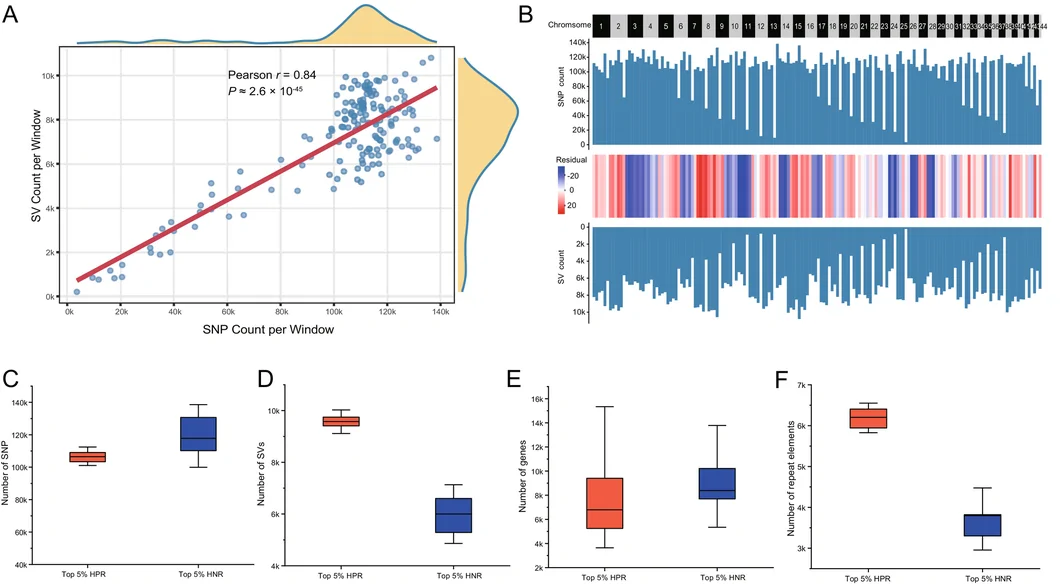
Comparison of whole‐genome sequencing (WGS)‐derived single nucleotide polymorphisms (SNPs) and structural variations (SVs) across the 44 chromosomes of *P. vannamei* . (A) Correlation between SNP and SV counts within 10‐Mb windows. (B) Residual plot from regressing SV density on SNP density within 10‐Mb windows, with chromosome labels shown above the plot. Positive residuals indicate regions with more SVs than expected, whereas negative residuals indicate fewer SVs than expected. The numbers of (C) SNPs, (D) SVs, (E) genes, and (F) repetitive elements within the top 5% HPR (high positive residuals) and top 5% HNR (high negative residuals) of windows after sorting residual values in descending order.

Genome annotation showed that 63.53% of all SVs overlapped repetitive sequence elements, a proportion higher than the repeat coverage of the reference genome. Among repeat classes, SSRs accounted for 27.35% of SV‐overlapping loci, DNA transposon repeats for 24.92%, and LINEs for 24.90% (Figure 1B). Deletions, duplications, and inversions showed broadly similar repeat‐overlap patterns, whereas insertions showed a relatively lower proportion of overlap with repetitive elements. Approximately 45% of insertions overlapped repetitive sequences, indicating that insertions were relatively more frequent in non‐repetitive regions compared with other SV types (Figure 1B). We further compared the genomic distribution of SVs with that of SNPs. A total of 16.68 million SNPs were identified across the 180 genomes after standard filtering. Genome‐wide, SNP and SV densities were highly correlated across genomic windows, with a Pearson correlation coefficient of 0.84 and a *p* value of approximately 2.6 × 10^−45^ (Figure 2A). To identify regions where SV density deviated from the SNP‐based expectation, we performed a regression analysis using SV count as the dependent variable and SNP count as the independent variable for each window, and then extracted the residuals (Figure 2B). The top 5% windows with high positive residuals (HPR) showed higher SV density than expected based on local SNP density, whereas the top 5% windows with high negative residuals (HNR) showed lower SV density than expected. HPR windows were enriched in TE and SSR contents and were mainly located in chromosome‐end regions, whereas HNR windows tended to overlap gene‐dense regions (Figure 2C–F).

### 3.2 Population Distribution of SV Polymorphisms

**FIGURE 3:**
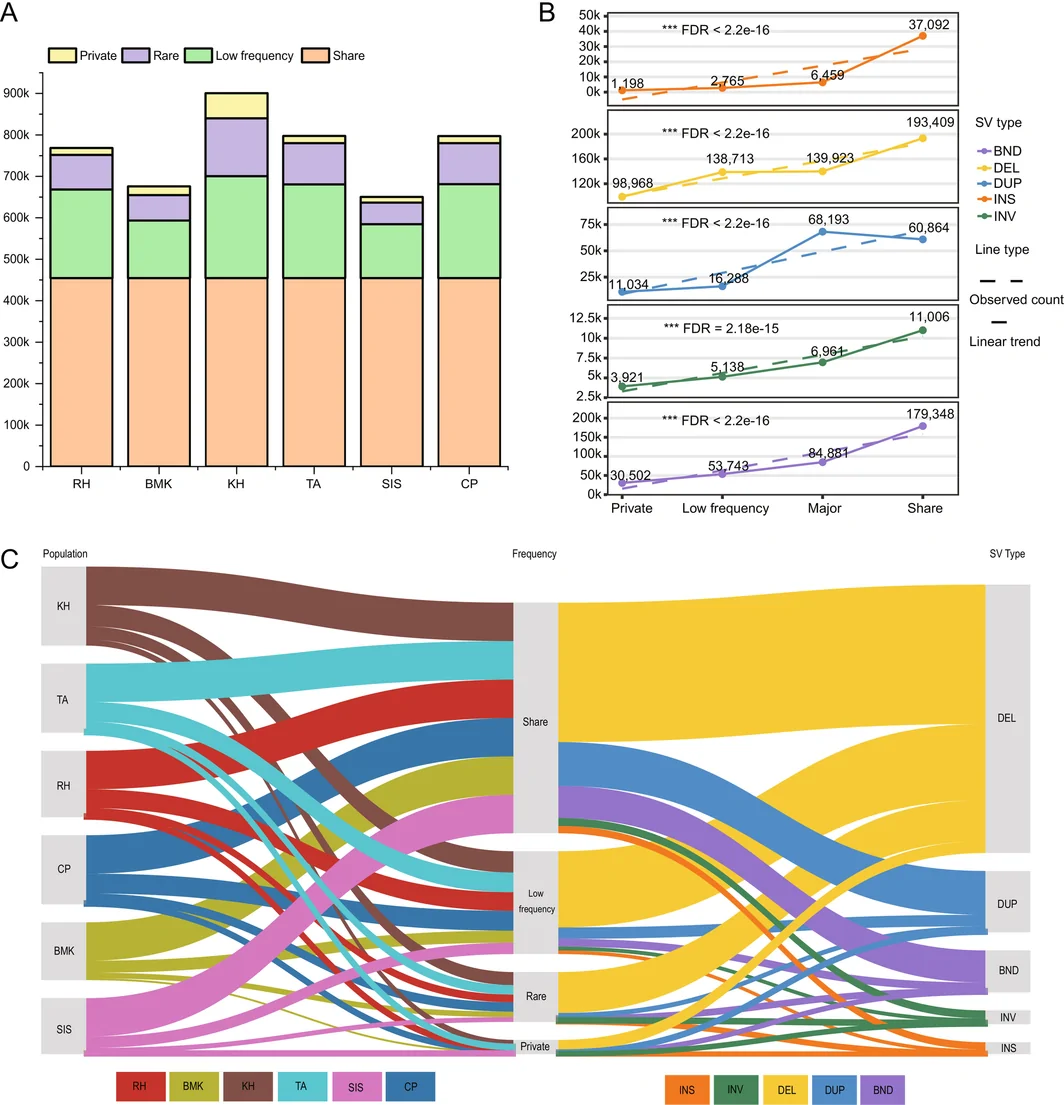
Composition of structural variations (SVs) across different populations. (A) The number of SVs of each type across the six populations, including shared SVs (detected in all populations), low‐frequency SVs (detected in 4–5 populations), rare SVs (detected in 2–3 populations), and private SVs (detected in only one population). (B) The number of SVs of different frequency categories in each population. (C) The number of SVs with different frequencies in the six populations and the composition of SV types within each frequency category.

We next examined the distribution of SV alleles among the six breeding populations. For each SV, its presence or absence was determined in each population. Based on the number of populations carrying an SV, variants were classified into four categories: shared SVs present in all 6 populations, low‐frequency SVs present in 4–5 populations, rare SVs present in 2–3 populations, and private SVs restricted to a single population. Among all 1,159,046 SVs, 481,719 SVs, accounting for approximately 41.56%, were shared by all six populations. A total of 306,417 SVs, accounting for approximately 26.4%, were classified as low‐frequency SVs, whereas 216,647 SVs, accounting for approximately 18.7%, were classified as rare SVs. Private SVs accounted for 145,623 SVs, corresponding to approximately 12.6% of all detected SVs (Figure 3A,C).

The composition of SV types differed among these frequency categories. Deletions showed an increasing proportion from shared SVs to private SVs, and this trend was statistically significant based on the Cochran–Armitage trend test (*p* < 2.2 × 10^−16^, FDR < 2.2 × 10^−16^) (Figure 3B). The proportion of private SVs also differed among populations. BMK and KH showed relatively higher proportions of private SVs, with values of 3.087% and 6.70%, respectively. In contrast, RH, TA, SIS, and CP showed lower and broadly similar proportions, with values of 2.156%, 2.164%, 2.104%, and 2.123%, respectively (Figure 3A,C).

### 3.3 Population Genetic Structure Inferred From SVs

To evaluate whether SVs can resolve population structure, we selected high‐confidence polymorphic SVs for population genetic analysis. After applying minor allele frequency and missing‐data filters, using MAF ≥ 0.05 and presence in at least 50% of individuals in at least one population, 295,542 SV loci were retained for downstream analysis.

**FIGURE 4:**
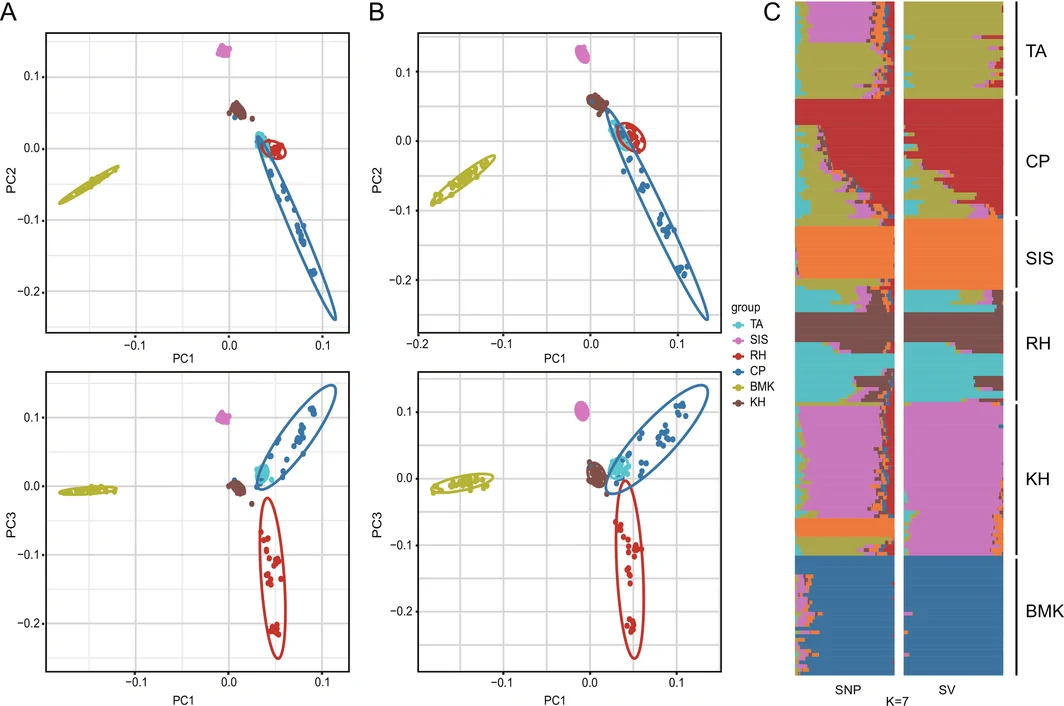
Whole‐genome SNP and SV analysis reveals population structure and genetic diversity in *P. vannamei* . (A) PCA plots of the first three principal components based on thinned SVs (*n* = 295,542). (B) PCA plots of the first three principal components based on thinned SNPs (*n* = 12,097). (C) Admixture plots for thinned SNPs (left, *n* = 12,097) and thinned SVs (right, *n* = 295,542) at *K* = 7.

Principal component analysis based on SV genotypes separated individuals according to their population origins (Figure 4A). In the first two principal components, samples were divided into three major clusters. BMK and SIS formed relatively distinct clusters, whereas RH, KH, TA, and CP clustered more closely together. This pattern was largely consistent with SNP‐based PCA results (Figure 4B). The first ten SV‐based principal components explained approximately 25% of the total variance (Figure S3A). Although RH, KH, TA, and CP were not fully separated by PC1 and PC2, PC3 further distinguished RH from the other three populations.

ADMIXTURE analysis was then performed using the SV genotype matrix. Cross‐validation analysis showed that the CV error reached its minimum at *K* = 7 (Figure 4C; Figures S3B and S4). At this *K* value, each population showed a dominant ancestry component, with minor shared components among some populations. RH individuals showed a mixed ancestry pattern, consistent with their known hybrid origin from the Miami and Oahu lines introduced to China (Figure 4C; Figure S4).

### 3.4 Signatures of Selection and Candidate Adaptive Genes

**FIGURE 5:**
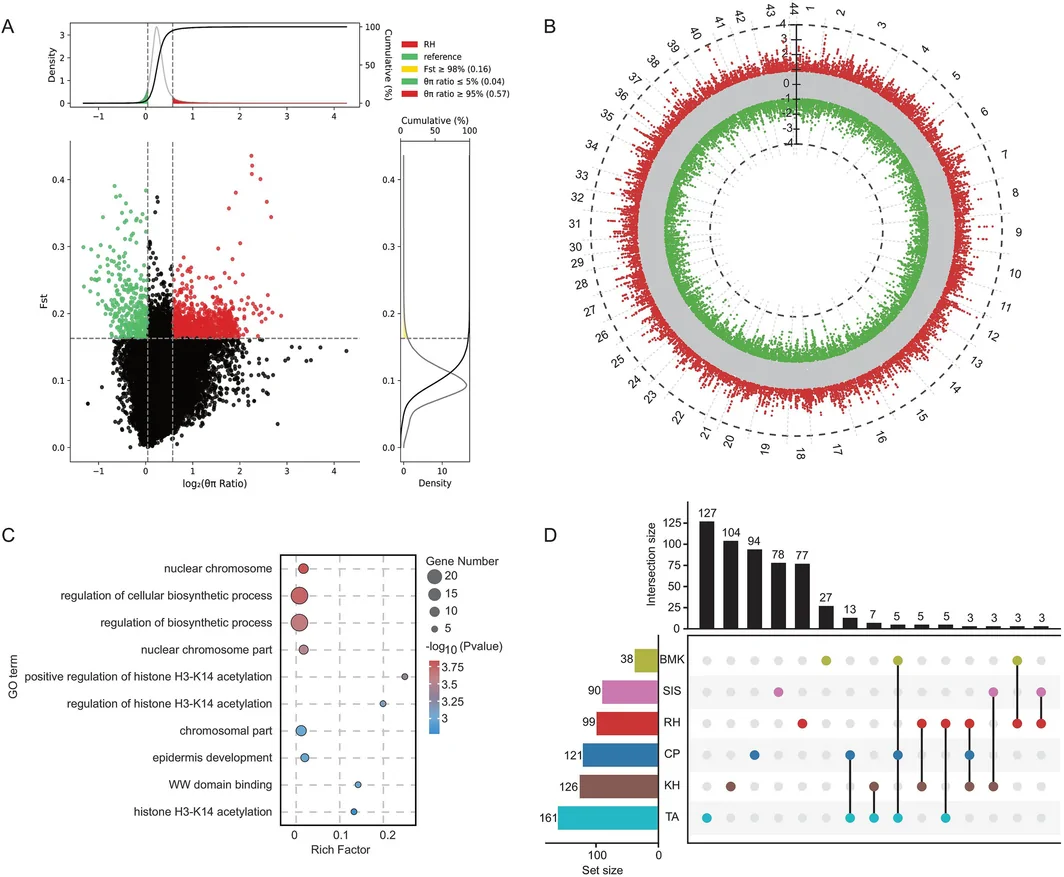
Population genetic differentiation and functional analysis of SVs. (A) Distribution of *F*_ST_ values and distribution of *θ*_*π*_ ratios (*θ*_*π*_ reference population/*θ*_*π*_ selective breed) calculated in 50 kb window with a 10 kb step in RH. Windows with the 5% largest or smallest of *θ*_*π*_ ratio and the top 2% largest *F*_ST_ were highlighted as regions under selection. (B) Distribution of mean XP‐EHH values calculated in 50 kb windows with a 10 kb step in RH. The top 10% of windows by XP‐EHH value were highlighted as candidate regions under selection. (C) Enriched GO terms of genes under selection in RH. (D) The sharing genes among groups.

To obtain candidate regions supported by additional population genetic evidence, we first performed selective sweep analysis based on population differentiation and nucleotide diversity. Two statistics were calculated in sliding windows of 50 kb with a 10 kb step size: the pairwise fixation index *F*_ST_ between the focal population and the other populations, and the nucleotide diversity ratio between the focal population and the pooled remaining populations. Based on the combined *F*_ST_ and *θ*_*π*_ ratio criteria, this approach identified 226, 181, 248, 274, 238, and 171 initial candidate selective sweep regions in RH, BMK, KH, TA, SIS, and CP, respectively (Figure 5A; Figures S5A–C and S6A,B, Table S3). Annotation of these regions identified 177, 123, 165, 238, 166, and 173 *F*_ST_–*θ*_*π*_ candidate genes in the corresponding populations (Figure 5A; Figures S5A–C and S6A,B, Table S4).

Then, we performed a genome‐wide haplotype‐based scan. This analysis identified population‐specific XP‐EHH candidate windows and corresponding annotated genes across the six groups (Figure 5B; Figures S5D–F and S6C,D, Tables S5 and S6). The number of windows and genes varied by population, with the highest counts observed in certain groups and the lowest in others, reflecting population‐level differences in selective sweep patterns.

To reduce false‐positive signals and identify more stringent candidate regions under selection, we intersected the XP‐EHH candidate windows with the initial *F*_ST_–*θ*_*π*_ candidate selective sweep regions. The overlapping regions were defined as high‐confidence candidate selected regions because they were jointly supported by haplotype‐based, population differentiation‐based, and nucleotide diversity‐based evidence. This analysis retained 150, 78, 189, 193, 162, and 113 high‐confidence candidate selected regions in RH, BMK, KH, TA, SIS, and CP, respectively. These regions accounted for 66.37%, 43.09%, 76.21%, 70.44%, 68.07%, and 66.08% of the initial *F*_ST_–*θ*_*π*_ candidate regions in the corresponding populations, indicating that a large proportion of differentiation‐ and diversity‐based sweep signals were also supported by XP‐EHH, particularly in KH and TA (Table 2; Table S7). Annotation of these high‐confidence regions identified 99, 38, 126, 161, 90, and 121 candidate genes in RH, BMK, KH, TA, SIS, and CP, respectively (Table S8).

**TABLE 2:** Summary of *F*_ST_–*θ*_*π*_ candidate selection windows and their overlap with XP‐EHH signals.

Methods	RH	BMK	KH	TA	SIS	CP
*F*_ST_–*θ*_*π*_	226	181	248	274	238	171
Overlapping with XP‐EHH	150	78	189	193	162	113
Overlap proportion with XP‐EHH	66.37%	43.09%	76.21%	70.44%	68.07%	66.08%

GO enrichment analysis revealed population‐specific functional patterns among candidate genes (Figure 5C; Figure S7). RH was mainly enriched for chromosome organization, biosynthetic‐process regulation, and histone H3‐K14 acetylation. BMK was enriched for omegasome, ER membrane complex, protein phosphorylation, histone H3‐K36 dimethylation, and developmental processes. KH showed enrichment in chromatin‐related terms, meiotic transcriptional regulation, DNA biosynthesis, and transcriptional repressor complexes. TA was mainly associated with omegasome membrane components, GTPase/EGO complex terms, ER protein folding, ARF signaling, and musculoskeletal movement. SIS was enriched for meiotic recombination, gene conversion, meiosis I regulation, nucleosome positioning, and GTP cyclohydrolase‐related activity. CP was mainly enriched for chitin‐based cuticle structure and development, extracellular matrix/space, and omegasome membrane components. These results suggest that selected regions in different populations may be associated with chromatin regulation, development and reproduction, membrane function, ER activity, and cuticle formation. The UpSet plot showed that most candidate selected genes were population‐specific, with limited overlap among populations. TA had the largest number of private genes, followed by KH, CP, SIS, RH, and BMK, indicating that selection signals were largely population‐specific. Only a few shared gene sets were detected among populations, with the main overlaps occurring between CP and TA, KH and TA, and BMK, CP and TA (Figure 5D).

Finally, we examined the relationship between selection signals and SVs. By integrating XP‐EHH candidate genes, *F*_ST_–*θ*_*π*_ candidate genes, and high‐confidence candidate selected regions, we identified 10 candidate genes carrying SV alleles in the corresponding selected populations (Table 3). Short deletions were particularly frequent among these variants. Some deletions were located within coding regions or near promoter regions, suggesting that these SVs may represent candidate variants for further functional validation.

**TABLE 3:** The SV events associated with genes under selection by *F*_ST_–*θ*_*π*_ and XP‐EHH.

Gene ID	Gene name	SV ID	SV type	SV length (bp)
RLOC_00005791	*Cht5*	DEL00090247	DEL	34
RLOC_00014585	*MAP3K7IP3 homolog*	DEL00430783	DEL	29
RLOC_00014586	*WFDC2*	—	—	—
RLOC_00014587	*EMC6*	—	—	—
RLOC_00014588	*PACS1*	—	—	—
RLOC_00014589	*UGT2A1*	—	—	—
RLOC_00017630	*PRDM14*	—	—	—
RLOC_00017631	*ZFX*	—	—	—
RLOC_00017632	*LLGL1*	DEL00559629	DEL	101
RLOC_00028543	*Tenascin‐like*	DUP01084921	DUP	154,466

## 4 Discussion

Our study provides the first comprehensive view of SVs in the Pacific white shrimp genome and shows that structural variation is closely shaped by the repeat‐rich architecture of this species' genome. A substantial proportion of SVs overlapped repetitive elements, exceeding the level expected from genome‐wide repeat coverage. This enrichment supports the view that repetitive sequences are major contributors to SV formation in *P. vannamei* (Yuan et al. 2021; Zhao et al. 2012). Similar patterns have been reported in other organisms, where repeats can promote structural mutations through transposon mobilization, non‐allelic homologous recombination, and replication slippage (Pande et al. 2025; Pascarella et al. 2022). The enrichment of SVs in chromosome‐end regions further suggests that local genome architecture influences SV accumulation. Our SNP–SV residual analysis strengthened this interpretation: the top 5% high positive residual windows were enriched in TE and SSR contents, whereas the top 5% high negative residual windows tended to overlap gene‐dense regions. These contrasting patterns suggest that SV distribution is shaped by both repeat‐associated mutational processes and negative selection against structural changes in functional genomic regions, consistent with observations in other species where SVs are often depleted near essential or functionally important genes (Ananda et al. 2013; Scott et al. 2021; Yuan et al. 2021; Zichner et al. 2013).

The population distribution of SVs provides additional insight into the breeding history and genetic differentiation of the six shrimp populations. Shared SVs likely represent ancestral structural polymorphisms retained across breeding lines, whereas private SVs may reflect population‐specific drift, founder effects, or subsequent breeding history (Jiao et al. 2025; Shah et al. 2025). The relatively higher proportions of private SVs in BMK and KH suggest that these two populations harbor more distinct structural variation than the other lines. Meanwhile, the increasing proportion of deletions from shared to private categories indicates that deletions may be more prone to population‐specific accumulation, although their functional consequences remain to be tested. Consistent with these patterns, SV‐based PCA and ADMIXTURE analyses recovered the major population relationships and broadly agreed with SNP‐based results reported previously for these populations (Wang et al. 2022). The mixed ancestry observed in RH also matched its known hybrid origin, indicating that SV markers can capture both divergence and admixture signals. Thus, SVs provide a useful complement to SNPs for population genomic analyses in shrimp breeding populations (Sui et al. 2024; Wang et al. 2022).

Selection‐scan analyses further revealed that genomic signatures of selection were largely distinct among populations. Rather than repeating the detailed GO enrichments described in the Results, the main implication is that different breeding lines appear to have been shaped by different selective pressures and genetic pathways (Sui et al. 2022, 2024; Wang et al. 2022). This is consistent with the complex nature of aquaculture breeding, in which growth, survival, reproduction, stress tolerance, and other traits may be emphasized differently across breeding programs (Max et al. 2025; Yin et al. 2025). Although a small number of candidate genes were detected across multiple populations, most signals were population‐specific, indicating limited recurrent selection at the same loci. The candidate genes were mainly associated with broad regulatory and cellular processes, including chromatin regulation, biosynthetic‐process regulation, histone modification, protein phosphorylation and modification, membrane‐associated functions, endoplasmic‐reticulum activity, meiotic transcriptional regulation, meiotic recombination, gene conversion, developmental and reproductive processes, extracellular structure, and chitin‐based cuticle formation. These functional categories may contribute to adaptive differentiation among breeding lines, but the candidate genes should still be interpreted cautiously because selection scans can be affected by demographic history, linkage, and window‐based resolution (Nam et al. 2017). By integrating XP‐EHH, *F*_ST_–*θ*_*π*_, and SV information, we prioritized selected regions that were supported by multiple lines of evidence and further connected these regions with structural variation. Several candidate genes carried SV alleles in the corresponding selected populations, with short deletions being particularly notable. Because some of these deletions were located within coding regions or near promoter regions, they may affect protein structure, functional domains, or cis‐regulatory sequences (Koeppel et al. 2024; Scott et al. 2021). However, this co‐localization does not demonstrate causality. These SVs should therefore be considered candidate variants for future genotype–phenotype association, expression analysis, and functional validation rather than confirmed causal mutations (Aygün et al. 2023; Gong et al. 2025).

From an applied perspective, the SV map and selected‐region candidates identified here provide a useful resource for future genome‐assisted breeding in *P. vannamei* . Population‐specific SVs may help trace breeding‐line differentiation, evaluate genetic background, and prioritize loci for trait association studies. The strong differentiation of selection signals among lines also suggests that different populations may contain complementary genetic resources, which could inform parental line selection and cross‐breeding design (Zenger et al. 2019). At the same time, these findings highlight the need to maintain broad genetic diversity and avoid excessive narrowing of the breeding base (Du et al. 2025). Overall, this study provides a genome‐wide SV resource and a set of candidates selected regions that can support future functional studies and marker‐assisted breeding in Pacific white shrimp.

## 5 Conclusion

This study provides the first genome‐wide characterization of structural variation in *P. vannamei* , identifying more than 1.1 million SVs associated with its repeat‐rich genomic architecture. SVs were strongly enriched in SSRs and transposable elements, indicating that their distribution is shaped by local genomic context and selective constraint in gene‐rich regions. Population‐level analyses demonstrated that SVs effectively capture genetic structure and differentiation among six major breeding lines, with BMK and KH showing the greatest accumulation of line‐specific variants. Selective sweep analyses further revealed mostly population‐specific candidate genes, reflecting divergence among breeding lines, while a small subset of recurrently selected genes carried short deletions with possible functional significance. Together, these results expand knowledge of structural genomic variation as a component of genetic diversity in domesticated shrimp and provide a useful resource for germplasm characterization. Importantly, SV‐based population genetic analyses may also provide molecular references for the selection of parental lines in hybrid breeding programs. Because these breeding populations have undergone long‐term artificial selection, recurrently selected SV‐associated loci may represent candidate genomic regions affected by breeding history and could serve as potential targets for future validation and marker development. More broadly, this study provides a foundation for further investigation of the functional effects of SVs on economically important traits in aquaculture species.

## Funding

This research was funded by National Key Research and Development Program of China (2021YFD1200805), National Natural Science Foundation of China (32373122), Hainan Yazhou Bay Seed Industry Laboratory ‘JBGS’ project (B24YQ0009), Hainan Province Science and Technology Talent Innovation Project (KJRC2025B14), Blue Seed Industry Innovation Project of Qingdao Institute of Blue Seed Industry (QDLYY‐2024014), and Fundamental Research Funds for the Central Universities (202341015).

## Conflicts of Interest

The authors declare no conflicts of interest.

## Supporting information


**Figure S1:** SNP‐based LD decay curve with bottom 5% annotated.

**Figure S2:** Length distributions of the four types of SVs, with those longer than 10 kb grouped together (A: INS, B: DEL, C: DUP, D: INV).

**Figure S3:** (A) Scree plot based on the PCA results of SVs. (B) Admixture cross‐validation error plots SNP and SV across *K* values 2–10.

**Figure S4:** Admixture plots based on SNP and SV for *K* values ranging from 2 to 10.

**Figure S5:** Population genetic differentiation of SVs. Distribution of *F*_ST_ values and Distribution of *θ*_*π*_ ratios (*θ*_*π*_ reference population/*θ*_*π*_ selective breed) calculated in 50 kb window with a 10 kb step. Windows with the 5% largest or smallest of *θ*_*π*_ ratio, and the top 2% largest *F*_ST_ were highlighted as regions under selection (A: BMK, B: KH, C: TA). Distribution of XP‐EHH values calculated in 50 kb windows with a 10 kb step. The top 10% of windows by XP‐EHH value were highlighted as candidate regions under selection (D: BMK, E: KH, F: TA).

**Figure S6:** Population genetic differentiation of SVs. Distribution of *F*_ST_ values and Distribution of *θ*_*π*_ ratios (*θ*_*π*_ reference population/*θ*_*π*_ selective breed) calculated in 50 kb window with a 10 kb step. Windows with the 5% largest or smallest of *θ*_*π*_ ratio, and the top 2% largest *F*_ST_ were highlighted as regions under selection (A: SIS, B: CP). Distribution of XP‐EHH values calculated in 50 kb windows with a 10 kb step. The top 10% of windows by XP‐EHH value were highlighted as candidate regions under selection (C: SIS, D: CP).

**Figure S7:** GO enrichment analysis of candidate genes under selection jointly identified by *F*_ST_, *θ*_*π*_, and XP‐EHH (A: BMK, B: KH, C: TA, D: SIS, E: CP).

**Table S1:** The numbers and retention rates of SNPs and SVs before and after filtering.

**Table S2:** SV length.

**Table S3:** Selected regions in each group by *F*_ST_–*θ*_*π*_.

**Table S4:** Selected genes in each group by *F*_ST_–*θ*_*π*_.

**Table S5:** Selected regions in each group by XP‐EHH.

**Table S6:** Selected genes in each group by XP‐EHH.

**Table S7:** Selected regions shared by *F*_ST_–*θ*_*π*_ and XP‐EHH.

**Table S8:** Genes annotated in selected regions shared by *F*_ST_–*θ*_*π*_ and XP‐EHH.

## Data Availability

The data presented in the study are deposited in the repository of figshare at https://doi.org/10.6084/m9.figshare.30745424.
