# Supplementary material for: Genome Wide Structural Variants Provide Insights Into Population Structure and Genetic Divergence in Pacific White Shrimp ( Penaeus vannamei ) Breeding Populations
Source: Evol Appl. 2026 Aug 24;19(8):e70312. doi: 10.1111/eva.70312 (PMC13504230; doi:10.1111/eva.70312)
Supplement: Supplementary file 1 [file EVA-19-e70312-s002.pdf]

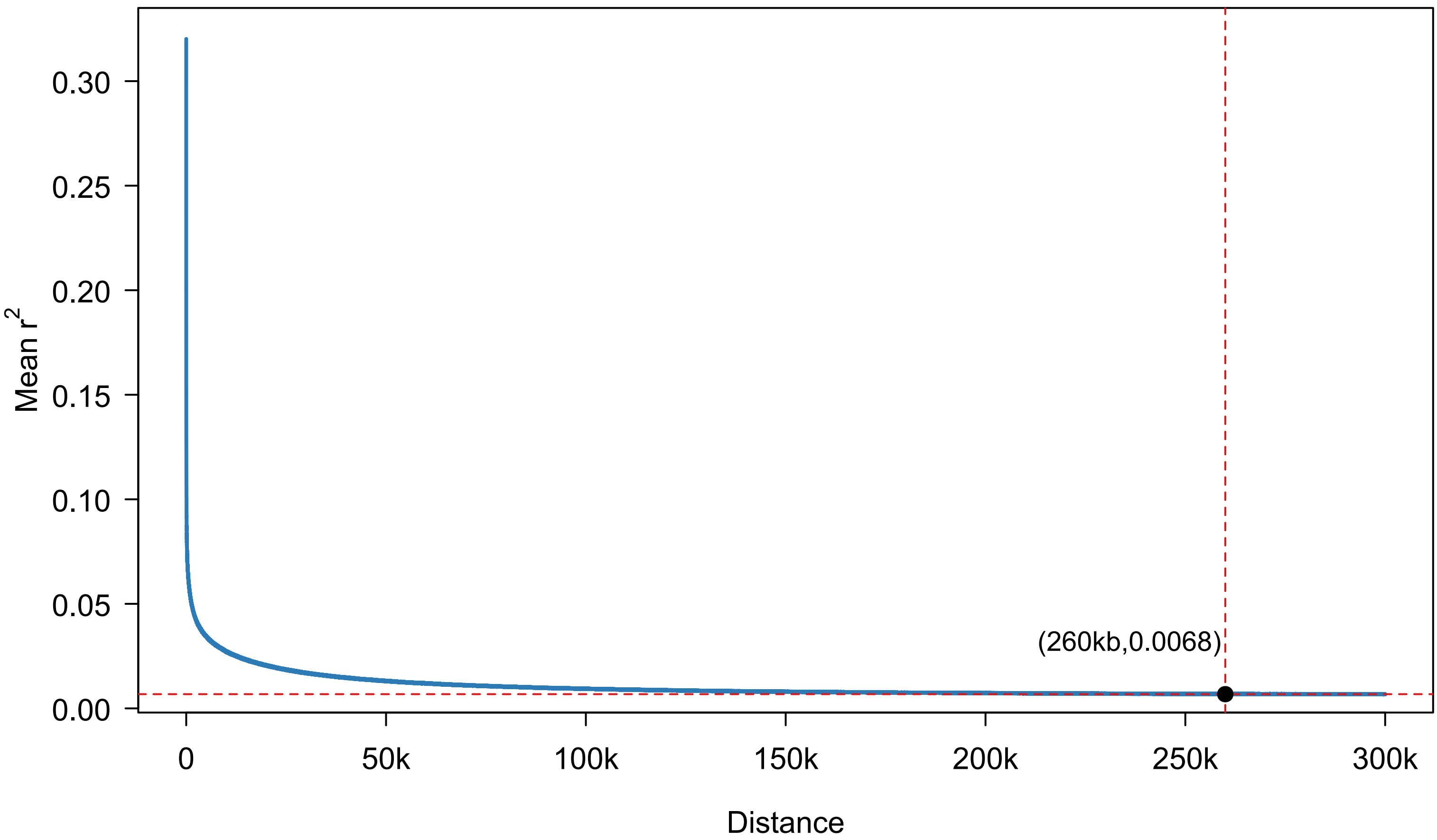

Mean r²
0.30
0.25
0.20
0.15
0.10
0.05
0.00
(260kb,0.0068)
0
50k
100k
150k
200k
250k
300k
Distance
