# Supplementary material for: Genome Wide Structural Variants Provide Insights Into Population Structure and Genetic Divergence in Pacific White Shrimp ( Penaeus vannamei ) Breeding Populations
Source: Evol Appl. 2026 Aug 24;19(8):e70312. doi: 10.1111/eva.70312 (PMC13504230; doi:10.1111/eva.70312)
Supplement: Supplementary file 2 [file EVA-19-e70312-s004.pdf]

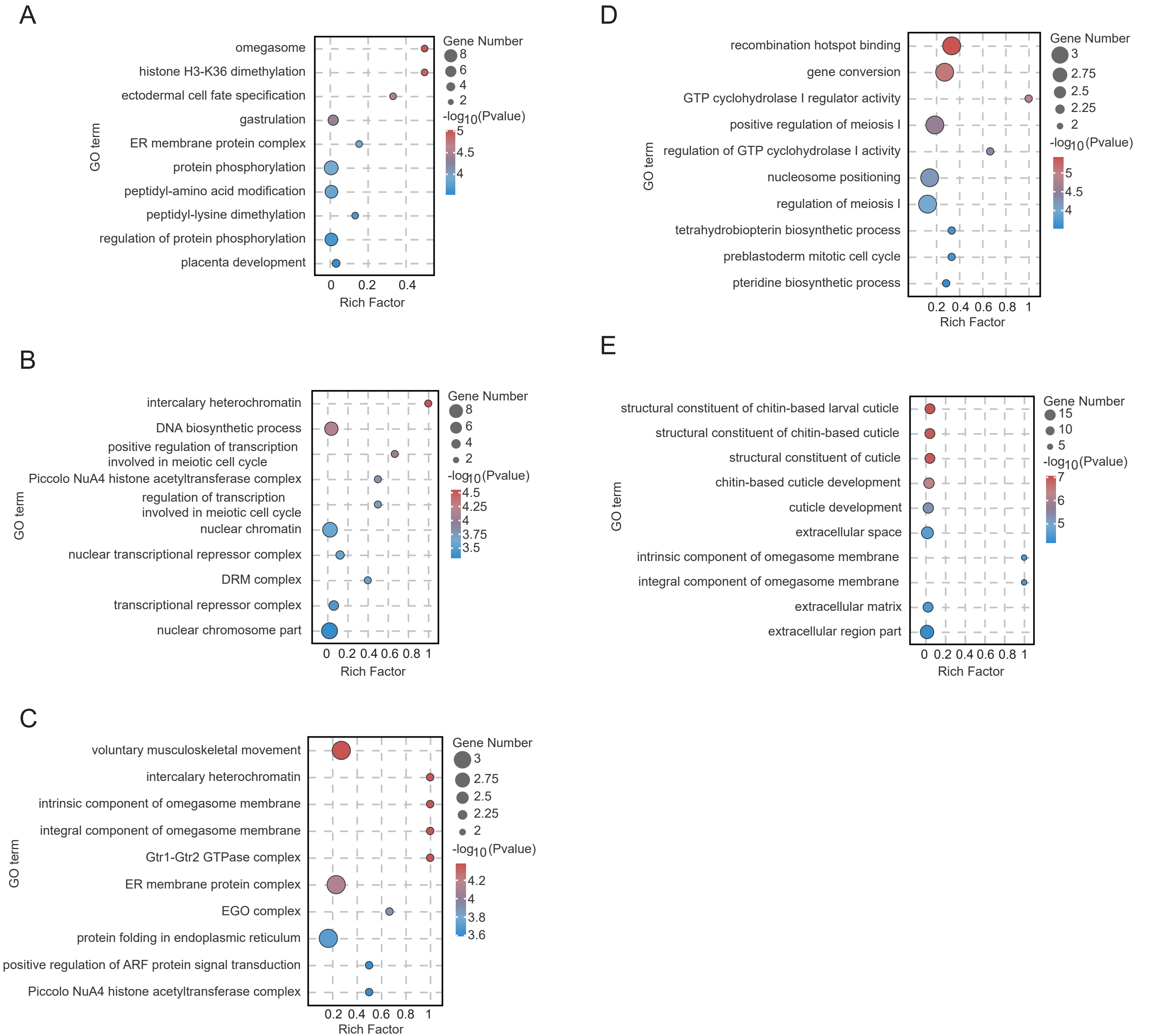
A
omegasome
histone H3-K36 dimethylation
ectodermal cell fate specification
gastrulation
ER membrane protein complex
protein phosphorylation
peptidyl-amino acid modification
peptidyl-lysine dimethylation
regulation of protein phosphorylation
placenta development
GO term
Rich Factor
Gene Number
-log10(Pvalue)
B
intercalary heterochromatin
DNA biosynthetic process
positive regulation of transcription involved in meiotic cell cycle
Piccolo NuA4 histone acetyltransferase complex
regulation of transcription involved in meiotic cell cycle
nuclear chromatin
nuclear transcriptional repressor complex
DRM complex
transcriptional repressor complex
nuclear chromosome part
GO term
Rich Factor
Gene Number
-log10(Pvalue)
C
voluntary musculoskeletal movement
intercalary heterochromatin
intrinsic component of omegasome membrane
integral component of omegasome membrane
Gtr1-Gtr2 GTPase complex
ER membrane protein complex
EGO complex
protein folding in endoplasmic reticulum
positive regulation of ARF protein signal transduction
Piccolo NuA4 histone acetyltransferase complex
GO term
Rich Factor
Gene Number
-log10(Pvalue)
D
recombination hotspot binding
gene conversion
GTP cyclohydrolase I regulator activity
positive regulation of meiosis I
regulation of GTP cyclohydrolase I activity
nucleosome positioning
regulation of meiosis I
tetrahydrobiopterin biosynthetic process
preblastoderm mitotic cell cycle
pteridine biosynthetic process
GO term
Rich Factor
Gene Number
-log10(Pvalue)
E
structural constituent of chitin-based larval cuticle
structural constituent of chitin-based cuticle
structural constituent of cuticle
chitin-based cuticle development
cuticle development
extracellular space
intrinsic component of omegasome membrane
integral component of omegasome membrane
extracellular matrix
extracellular region part
GO term
Rich Factor
Gene Number
-log10(Pvalue)
