# Supplementary material for: Genome Wide Structural Variants Provide Insights Into Population Structure and Genetic Divergence in Pacific White Shrimp ( Penaeus vannamei ) Breeding Populations
Source: Evol Appl. 2026 Aug 24;19(8):e70312. doi: 10.1111/eva.70312 (PMC13504230; doi:10.1111/eva.70312)
Supplement: Supplementary file 3 [file EVA-19-e70312-s007.pdf]

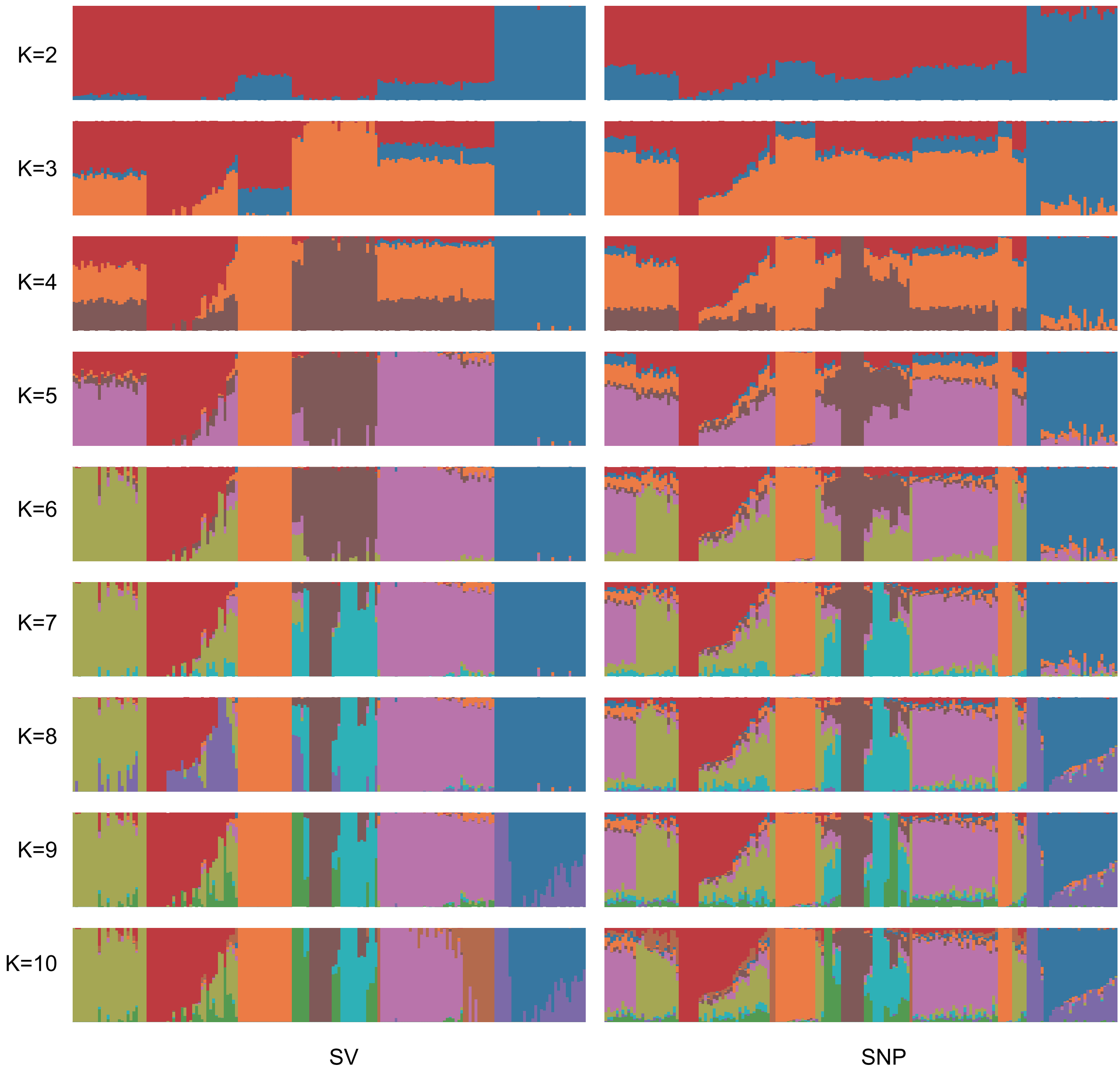
K=2
K=3
K=4
K=5
K=6
K=7
K=8
K=9
K=10
SV
SNP
