# Supplementary material for: Genome Wide Structural Variants Provide Insights Into Population Structure and Genetic Divergence in Pacific White Shrimp ( Penaeus vannamei ) Breeding Populations
Source: Evol Appl. 2026 Aug 24;19(8):e70312. doi: 10.1111/eva.70312 (PMC13504230; doi:10.1111/eva.70312)
Supplement: Supplementary file 4 [file EVA-19-e70312-s008.pdf]

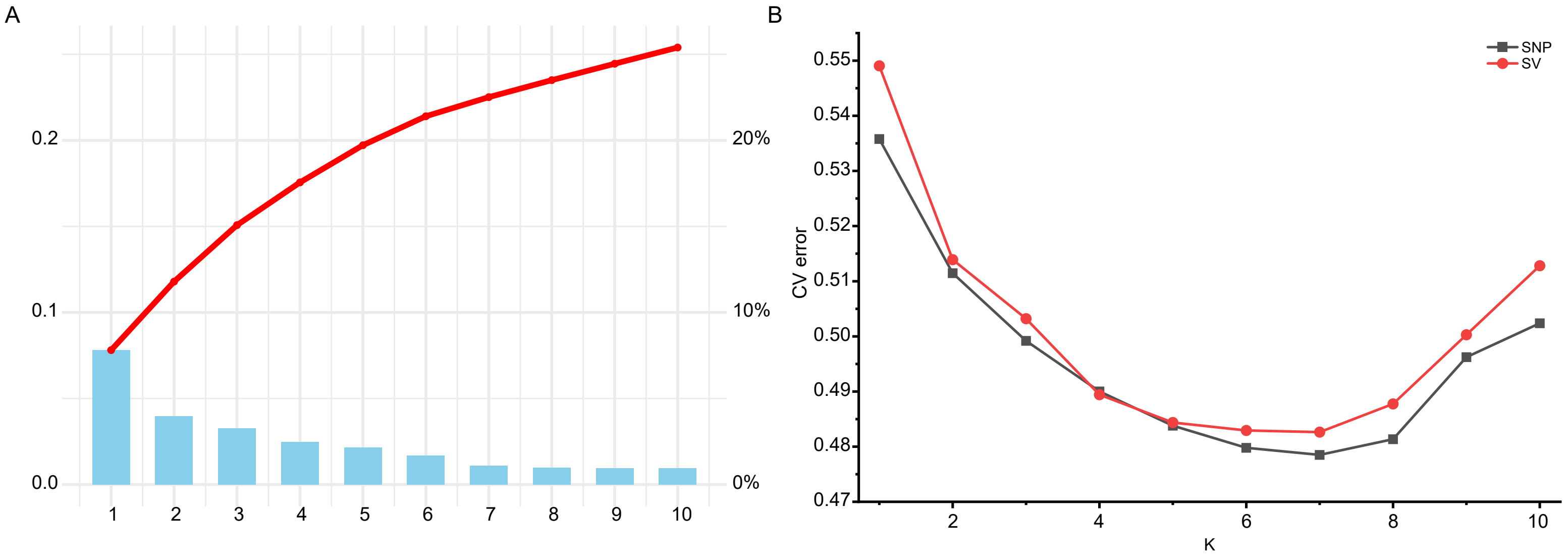

A
0.2
0.1
0.0
20%
10%
0%
1
2
3
4
5
6
7
8
9
10
B
SNP
SV
CV error
0.55
0.54
0.53
0.52
0.51
0.50
0.49
0.48
0.47
2
4
6
8
10
K
