# Supplementary material for: Genome Wide Structural Variants Provide Insights Into Population Structure and Genetic Divergence in Pacific White Shrimp ( Penaeus vannamei ) Breeding Populations
Source: Evol Appl. 2026 Aug 24;19(8):e70312. doi: 10.1111/eva.70312 (PMC13504230; doi:10.1111/eva.70312)
Supplement: Supplementary file 5 [file EVA-19-e70312-s011.pdf]

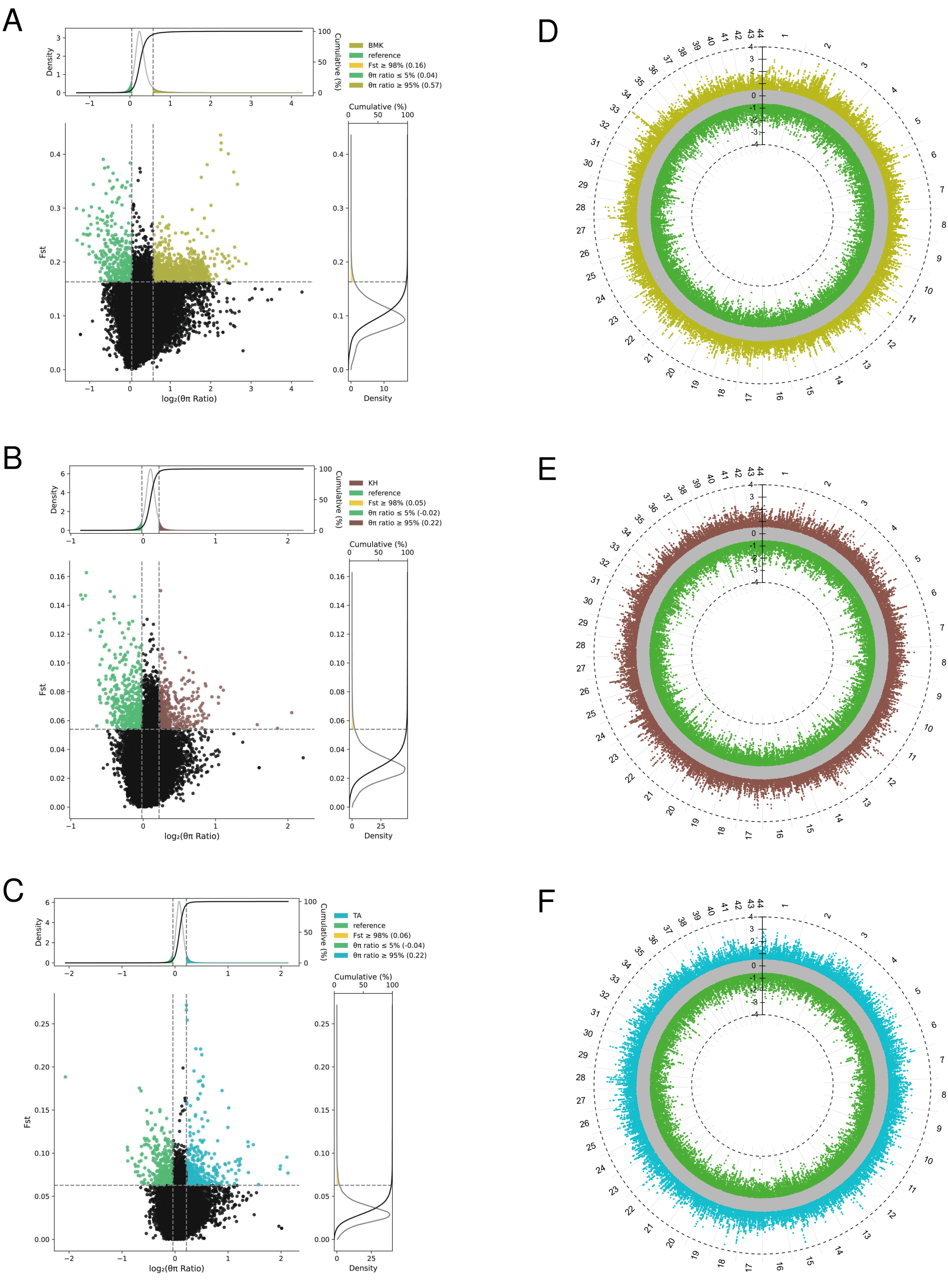
A
BMK
reference
Fst ≥ 98% (0.16)
θπ ratio ≤ 5% (0.04)
θπ ratio ≥ 95% (0.57)
Density
Cumulative (%)
Fst
log₂(θπ Ratio)
B
KH
reference
Fst ≥ 98% (0.05)
θπ ratio ≤ 5% (-0.02)
θπ ratio ≥ 95% (0.22)
Density
Cumulative (%)
Fst
log₂(θπ Ratio)
C
TA
reference
Fst ≥ 98% (0.06)
θπ ratio ≤ 5% (-0.04)
θπ ratio ≥ 95% (0.22)
Density
Cumulative (%)
Fst
log₂(θπ Ratio)
D
E
F
