# Supplementary material for: Genome Wide Structural Variants Provide Insights Into Population Structure and Genetic Divergence in Pacific White Shrimp ( Penaeus vannamei ) Breeding Populations
Source: Evol Appl. 2026 Aug 24;19(8):e70312. doi: 10.1111/eva.70312 (PMC13504230; doi:10.1111/eva.70312)
Supplement: Supplementary file 6 [file EVA-19-e70312-s012.pdf]

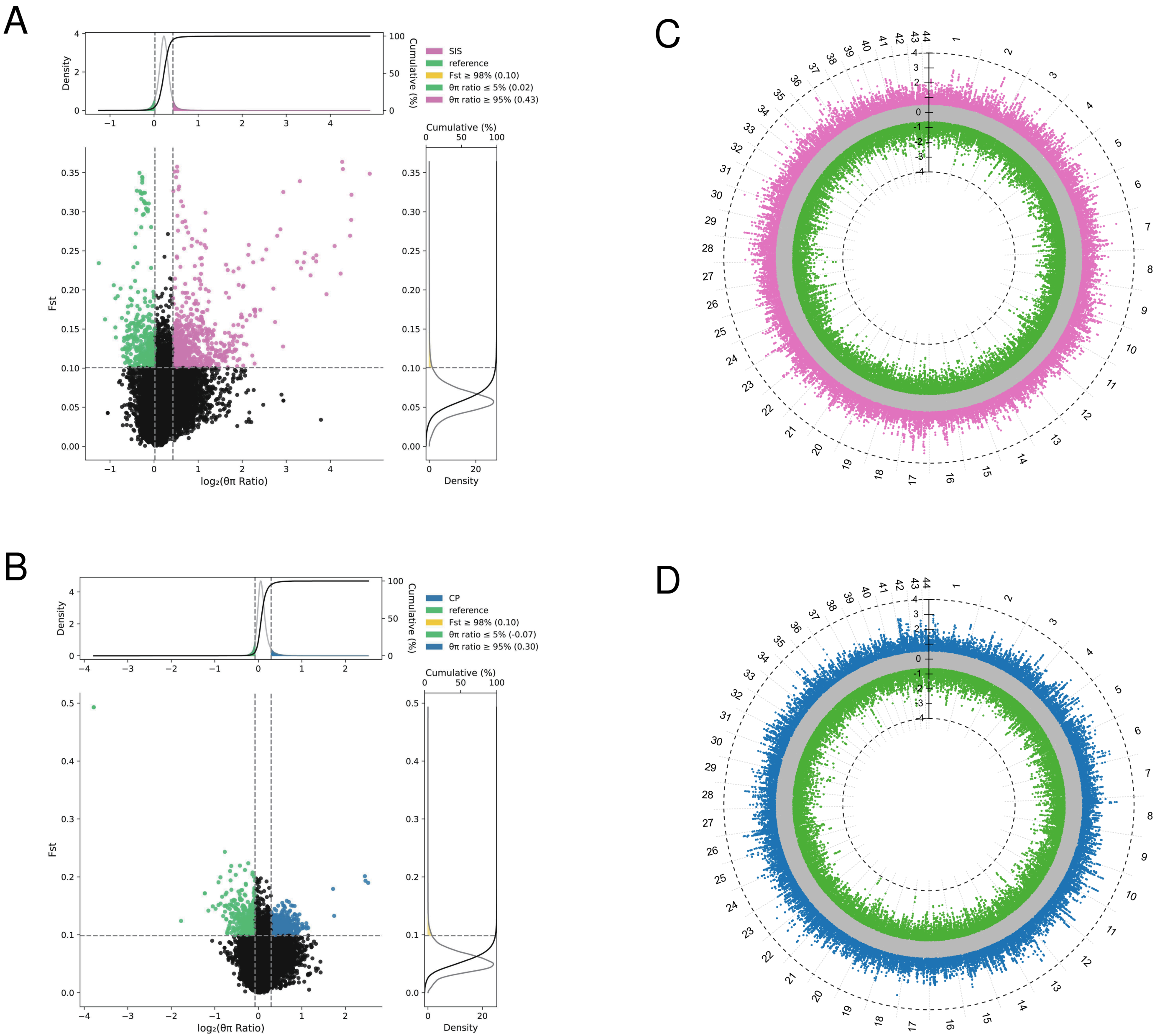
A
B
C
D
SIS
reference
Fst ≥ 98% (0.10)
θπ ratio ≤ 5% (0.02)
θπ ratio ≥ 95% (0.43)
CP
reference
Fst ≥ 98% (0.10)
θπ ratio ≤ 5% (-0.07)
θπ ratio ≥ 95% (0.30)
Density
Cumulative (%)
Fst
log₂(θπ Ratio)
