# Supplementary material for: Genome Wide Structural Variants Provide Insights Into Population Structure and Genetic Divergence in Pacific White Shrimp ( Penaeus vannamei ) Breeding Populations
Source: Evol Appl. 2026 Aug 24;19(8):e70312. doi: 10.1111/eva.70312 (PMC13504230; doi:10.1111/eva.70312)
Supplement: Supplementary file 7 [file EVA-19-e70312-s014.pdf]

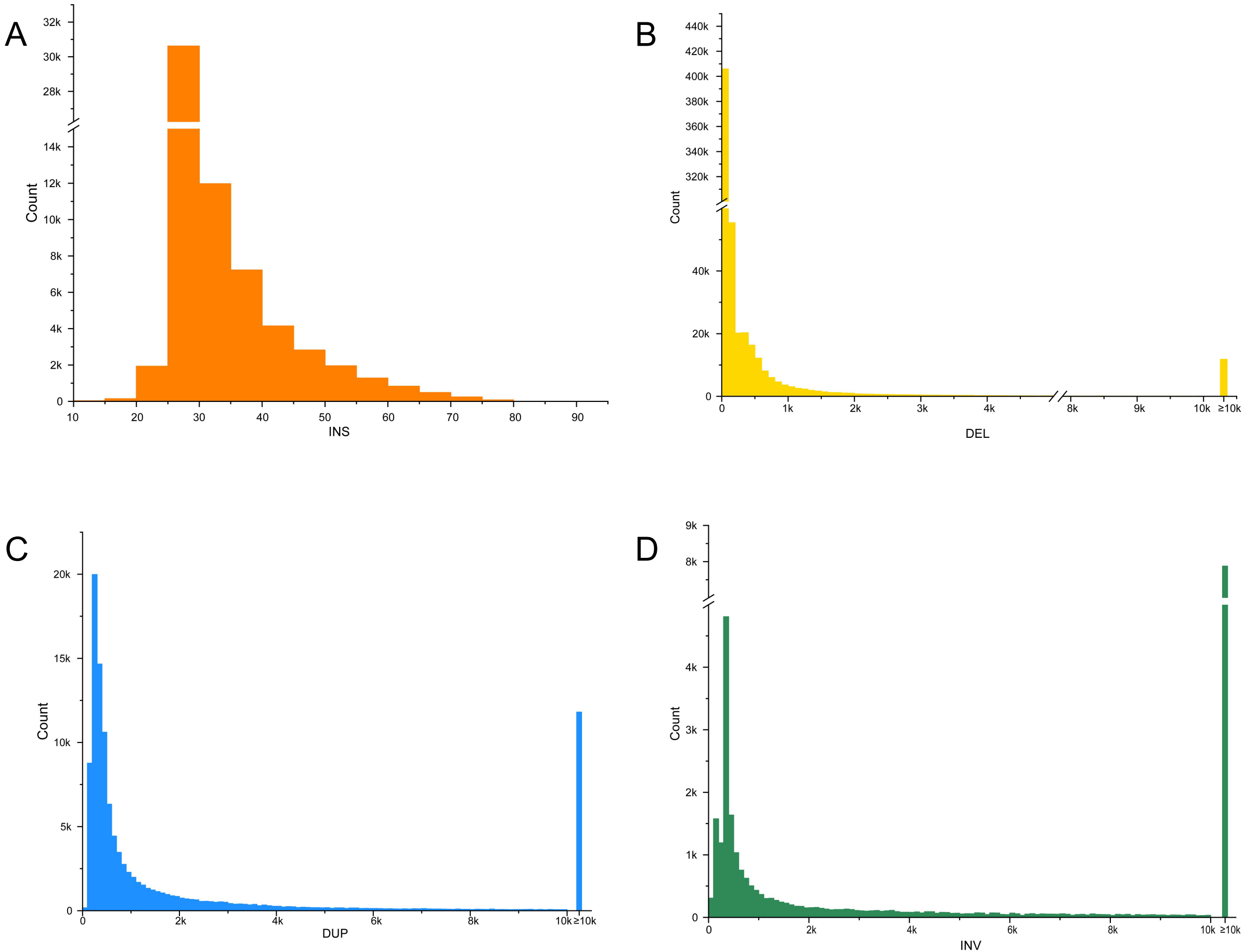

A
Count
INS
B
Count
DEL
C
Count
DUP
D
Count
INV
